# PolyGEE: a generalized estimating equation approach to the efficient and robust estimation of polygenic effects in large-scale association studies

**DOI:** 10.1093/biostatistics/kxx040

**Published:** 2017-08-31

**Authors:** Julian Hecker, Dmitry Prokopenko, Christoph Lange, Heide Loehlein Fier

**Affiliations:** 1 Department of Biostatistics, Harvard T.H. Chan School of Public Health, 655 Huntington Avenue, Boston, MA 02115, USA and Department of Genomic Mathematics, University of Bonn, Sigmund-Freud-Strasse 25, 53127 Bonn, Germany; 2 Channing Division of Network Medicine, Brigham and Women’s Hospital, 181 Longwood Avenue, Boston, MA 02115, USA; 3 Department of Biostatistics, Harvard T.H. Chan School of Public Health, 655 Huntington Avenue, Boston, MA 02115, USA and Channing Division of Network Medicine, Brigham and Women’s Hospital, 181 Longwood Avenue, Boston, MA 02115, USA

**Keywords:** Family-based association studies, GEE, GWAS, Polygenic effects, Summary statistics

## Abstract

To quantify polygenic effects, i.e. undetected genetic effects, in large-scale association studies, we propose a generalized estimating equation (GEE) based estimation framework. We develop a marginal model for single-variant association test statistics of complex diseases that generalizes existing approaches such as LD Score regression and that is applicable to population-based designs, to family-based designs or to arbitrary combinations of both. We extend the standard GEE approach so that the parameters of the proposed marginal model can be estimated based on working-correlation/linkage-disequilibrium (LD) matrices from external reference panels. Our method achieves substantial efficiency gains over standard approaches, while it is robust against misspecification of the LD structure, i.e. the LD structure of the reference panel can differ substantially from the true LD structure in the study population. In simulation studies and in applications to population-based and family-based studies, we illustrate the features of the proposed GEE framework. Our results suggest that our approach can be up to 100% more efficient than existing methodology.

## 1. Introduction

While genome-wide association studies (GWAS) led to the discovery of many genetic risk loci for complex diseases and traits, the overall magnitude of the combined genetic effects on the disease phenotype of interest that is explained by GWAS findings is much smaller than classical heritability studies suggested (Manolio *and others*, 2008). This motivated large-scale efforts, e.g. Psychiatric Genomics Consortium (PGC), to combine as many available GWAS as possible for any particular disease and trait, and perform meta-analyses across all available studies. The large-scale efforts identified additional disease loci, but the overall genetic effect that is attributable to all significant GWAS signals still remains substantially smaller than the estimated heritability.

At the same time, in many of the meta-analyses and large-scale GWAS, a genomic control factor (Devlin *and others*, 2001) (global inflation factor of the association test statistics) that is clearly greater than one was observed, raising concerns about the validity of the statistical analysis. The inflation of the association test statistics can be explained by differences in terms of ancestry and/or phenotypic characteristics between the study populations of the meta-analysis. Such differences would lead to study heterogeneity which is difficult to account for in such analysis. Current efforts to maximize sample size by including as many studies as possible in the meta-analysis could amplify this effect. An alternative explanation for the observed inflation of association test statistics in such large-scale analyses is that the inflation of the genomic control factor is caused by large numbers of true positive association signals that do not formally reach the level of genome-wide significance (polygenic effects). While initial simulation studies suggest that polygenic effects can be a plausible explanation for such inflations of the genomic control factor (Devlin *and others*, 2001), statistical methodology is required to address this important research question.

For population-based studies of unrelated individuals, *LD Score regression* (Bulik-Sullivan *and others*, 2015b) proposed a marginal mean model for the single-variant association test statistics and applied a weighted linear regression technique to estimate the corresponding model parameters, quantifying the amount of population stratification and polygenic effects. Standard errors for the estimates are obtained through a jackknife procedure, as classical regression assumptions are violated due to the complex correlation structure between association test statistics.

Here, we derive a general marginal model for single-variant association test statistics of complex diseases and traits in large-scale studies that is valid for arbitrary study designs, e.g. population-based studies, family-based studies or any combination of those. For the special case of population-based association test statistics, our general mean model is equivalent to the mean model of LD Score regression. For the general mean model, we propose an efficient and robust framework to estimate the parameters of the marginal model. Our approach extends the idea of GEEs and utilizes LD matrices estimated from external reference panels/populations. If the LD matrices from the reference panels approximate the true correlation structure of test statistics reasonably well, our framework achieves a substantially increased efficiency over LD Score regression estimation. In contrast to existing methods as VEGAS (Liu *and others*, 2010) or ImpG (Pasaniuc *and others*, 2014), our framework does not require that external LD matrices exactly match the correlation structure of the study and is therefore robust against LD misspecification due to population differences or small sample sizes of reference panels. Furthermore, our GEE based approach provides asymptotic valid standard errors, making simulation or bootstrap methods redundant. In simulation studies, we examine the efficiency of our approach and its robustness against misspecification of the LD structure. In application to the population-based studies of the PGC, we observe an efficiency increase compared to LD Score regression. We also illustrate the approach by an application to a family-based association study for autism.

## 2. Methods

In this section, we derive a flexible marginal model for association test statistics in large-scale association studies. Based on that, we define the PolyGEE framework for the estimation of the polygenic effects.

### 2.1. General marginal model for association test statistics of complex diseases

For the complex trait of interest, we want to estimate the amount of polygenic effects at a genome-wide level using a dense set of $ n_{\text{SNPs}}$ common SNPs that are ordered by their physical location along the chromosomes. For these loci, the corresponding summary association test statistics are denoted by $ \chi^{2}_1,\dots,\chi^{2}_{n_{\rm SNPs}}$. They typically are obtained from a population-based study, a family-based study or from a meta-analysis/combination of both study designs. For a population-based design, the association test statistics are often score tests and, in family-based designs, FBAT statistics are frequently used (Lake *and others*, 2001; Laird *and Lange*, 2010). We propose the following general marginal mean model for association test statistics

(2.1) \begin{equation*} \mathbf{E}[\chi^{2}_l]=\beta_1+C_{\text{study}} l_l \beta_2, \quad l=1,\dots,n_{\text{SNPs}}, \label{mean} \end{equation*}

where the parameter $ l_l$ denotes the LD Score of variant $ l$. The LD Score (Bulik-Sullivan *and others*, 2015b) $ l_l= \sum\limits_{v} r^{2}_{lv}$ measures the total amount of LD between the variant $ l$ and variants within a genetic distance of 1 centimorgan (cM). Bulik-Sullivan *and others* (2015b) observed that this genetic distance is sufficient to describe the LD structure locally. In this summation, $ r^{2}_{lv}$ denotes the LD measure $ r^{2}$ between variant $ l$ and a variant $ v$.

To identify and quantify the different sources of inflation, we include two parameters in equation (2.1). The first parameter $ \beta_1$ measures the amount of global population stratification in the study that causes the inflation of the test association statistics at a genome-wide level. The second parameter $ \beta_2$ measures the overall polygenic effects. We will see that that the second parameter can be linked to the heritability of the disease/trait. The specification of the constant $ C_{\rm study}$ depends only on the design of the association study by a priori-known study parameters such as sample size, disease prevalence etc. Below, we describe that the already known mean models for case-control and quantitative trait studies of unrelated individuals are special cases of equation (2.1) and derive the study parameter $ C_{\rm study}$ for these scenarios. Furthermore, for family-based association studies with dichotomous traits, we derive the corresponding special case of equation (2.1) and, based on these findings, we can derive the parameter $ C_{\rm study}$ for the more general case of association studies with mixed designs, i.e. combinations of population-based and family-based designs.

The variance of association test statistic $ \chi^{2}_l$ in our general marginal model is described by

(2.2) \begin{equation*} \text{Var}(\chi^{2}_l )= \phi (\mathbf{E}[\chi^{2}_l ] )^{2}, \label{variance} \end{equation*}

where $ \phi$ is a dispersion parameter. In 2.1.4, we argue why equation (2.2) is a reasonable assumption. To understand the special cases of the general marginal model for population- and family-based study designs for dichotomous traits, we first establish the connection between the relative risk and the liability model.

#### 2.1.1. Link between relative risk and explained variance.

For a complex disease with prevalence $ K$ and under the assumption of a multiplicative relative risk model, the phenotypic variance explained by a causal variant, here denoted by $ q^{2}$, with a minor allele frequency (MAF) $ p$ on the liability scale is approximately given by (Peyrot *and others*, 2016; Yang *and others*, 2011b)

(2.3) \begin{equation*} q^{2}= \frac{2p(1-p) (\lambda-1)^{2}}{c^2}, \label{liability} \end{equation*}

where the parameter $ \lambda$ denotes the relative risk of the minor allele and $ c=\frac{t}{K}$ is a scaling factor. The parameter $ t$ is the height of the standard normal curve at the point of the $ 1-K$ quantile. Equation (2.3) is the key to connect the mean of an association test statistic, which clearly depends on the relative risk $ \lambda$ and the MAF $ p$, to the variance explained by the causal variant. According to the established literature, the heritability $ h^2$ is now defined as the sum of explained variances of causal variants, assuming additive effects and equal effect size distributions. The heritability defined in this way measures the total amount of polygenic effects, this explains why we can identify $ \beta_2$ with $ h^{2}$ under these assumptions.

#### 2.1.2. Population-based association studies.

In the case of a case-control study, our marginal mean model is defined by the LD Score regression mean model for the association test statistics $ \chi^{2}_l$, given by (Bulik-Sullivan *and others*, 2015b)

(2.4) \begin{equation*} \mathbf{E} [\chi^{2}_l]=1+Na+l_l \frac{n_{\rm cases} n_{\rm controls} c^2 h^2}{(1-K)^2 NM}, \label{casecontrol} \end{equation*}

where we denote the sample size by $ N$, the average heritability per SNP by $ \frac{h^2}{M}$, the numbers of cases, respectively, controls by $ n_{\rm cases}$, respectively, $ n_{\rm controls}$, and the amount of population stratification by $ a$. The characterization of $ \frac{h^2}{M}$ requires the specification of the number of SNPs $ M$ that is used in the computation of the LD Scores. To avoid an overestimation of the heritability, $ M$ is restricted to the number of common SNPs with a MAF above (Bulik-Sullivan *and others*, 2015a) 5%, as suggested by LD Score regression. For unascertained studies with quantitative traits, the mean model is given by (Bulik-Sullivan *and others*, 2015b)

(2.5) \begin{equation*} \mathbf{E}[\chi^{2}_l ]=1+Na+l_l \frac{Nh^2}{M}. \label{quant} \end{equation*}

Equations (2.4) and (2.5) are linear approximations of the mean of association statistics. These formulas can be derived using a Taylor approximation of the non-centrality parameter (NCP) (Yang *and others*, 2011b) with respect to the relative risk $ \lambda$ (utilizing the small effect size assumption of a polygenic architecture), applying equation (2.3), using the definition of the LD Scores $ l_l$ and assuming constant variances of causal variants that are uniformly distributed along the genome.

#### 2.1.3. Family-based case-control studies.

We consider a large-scale family-based association study with $ N$ independent nuclear families, all families are assumed to be of the same type, e.g. the same number of affected or unaffected offspring ($ n_a$ and $ n_u$, respectively), and with parental genotype information available. We relax this assumption later. We assume that transmissions to offspring are independent given the parental genotypes. We assume that the variant $ l$ is tested by the general association test approach which is implemented in FBAT (Lake *and others*, 2001; Lange *and Laird*, 2002). We denote the offset parameter by $ z$. Based on a second order Taylor approximation in $ \lambda$ around $ \lambda=1$, we find the following expression for the expectation value of the squared test statistic $ \chi^{2}_l$.

Proposition 1 (2.6) \begin{equation*} \mathbf{E}[ \chi^{2}_l ] \approx 1 + r^{2}_{l\text{DSL}} \frac{ c^{2} \frac{1}{2} N((1-z) n_a+z \frac{K}{(1-K)} n_u )^2}{(1-z)^2 n_a+z^2 n_u } q^{2}_{\rm DSL}, \label{fambased} \end{equation*}

where $ q^{2}_{\rm DSL}$ denotes the explained variance of a causal variant in LD $ r^{2}_{l\text{DSL}}$ with variant $ l$. The parameters $ N$, $ z$, $ n_a$ and $ n_u$ are defined as above, the parameter $ K$ denotes the prevalence of the disease.

The derivation of this approximation can be found in Appendix A of the supplementary materials available at *Biostatistics* online. In the following, analogously to the population-based scenario, we consider the approximation as strict equality to model the mean. Furthermore, following the arguments as in the setting of population-based studies (constant variances of causal variants, uniform spatial distribution along the genome), we can state

(2.7) \begin{equation*} \mathbf{E}[\chi^{2}_l ]=1+Na+l_l \frac{ c^{2} \frac{1}{2} N((1-z) n_a+z K/(1-K) n_u )^2}{(1-z)^2 n_a+z^2 n_u} \frac{h^{2}}{M} . \label{fambased2} \end{equation*}

The extension of the mean model to multiply family types is straightforward (Appendix A.2 of the supplementary materials available at *Biostatistics* online). Equation (2.6) characterizes the approximate power of family-based studies for all combinations of affected and unaffected offspring with reference to small effect sizes (small relative risks). Our derivation thereby does not rely on an explicit analytic formula for the exact NCP. The latter is only known for some special cases as trios and sibling pairs (Deng and Chen, 2001; Knapp, 1999). Our results are in line with corresponding second order Taylor approximations of these NCPs in $ \lambda$ around $ \lambda=1$.

#### 2.1.4. Equation (2.2) and $ C_{\rm study}.$

Obviously, equations (2.4), (2.5), and (2.7) are covered by model equation (2.1) with an appropriate choice for $ C_{\rm study}$ and identifying $ \beta_1$ with $ 1+Na$ and $ \beta_2$ with $ h^{2}$. For a meta-analysis of population- and family-based designs, $ C_{\rm study}$ is constructed by the corresponding weighted sum.

If we utilize the assumption of normally distributed genetic effects for the moment, we can conclude that the equation (2.2) is valid with $ \phi=2$. The assumption of normally distributed effects was used in both the derivation of the linear mixed model of GCTA (Yang *and others*, 2011a) and the motivation for the heteroscedasticity weights of the LD Score regression (Bulik-Sullivan *and others*, 2015b). We relax assumption equation (2.2) by assuming an arbitrary nuisance parameter $ \phi$.

### 2.2. PolyGEE framework

Now, we propose our PolyGEE approach to estimate the parameters $ \beta_1$ and $ \beta_2$ from the given set of association test statistics. The correlation structure of the test statistics is mainly caused by the LD structure between the corresponding genetic variants. Since meaningful degrees of LD around a variant usually do not exceed beyond 1 cM (Laird and Lange, 2010), we can assume here a band correlation structure. LD between two loci can be estimated from reference panels such as the 1000 Genomes Project (1000 Genomes Project Consortium *and others*, 2012) for specific subpopulations. These LD matrices estimated from reference panels provide reasonable approximations of the correlation structure of our study data for a moderate-sized number of variants. However, they cannot be assumed to be completely correct, due to subpopulation differences, small reference panel sample sizes, genetic effects or covariate adjustment (Xu *and others*, 2015). We extend the GEE approach to estimate the parameters $ \beta_1$ and $ \beta_2$ in the mean model for the association test statistics (2.1). GEE approaches provide unbiased estimates for statistical models that are specified only by their first and second moments (Liang and Zeger, 1986). The observations are grouped into clusters that are assumed to be statistically independent and, within the clusters, so-called working-correlation matrices are specified which are supposed to approximate the true correlation structure. Even, if the correlation matrices are severely mis-specified, the GEE approach is robust against this model violation and provides both consistent estimates of the parameters of the mean model and valid standard errors. However, the efficiency of the GEE approach benefits from an accurate specification of the correlation structure. Note that the quantity $ l_l$ as the average of LD measures can be estimated more robustly from reference panels than high-dimensional LD matrices. Therefore, we assume the general marginal model to be correctly specified. To apply the GEE methodology to our model (2.1 and 2.2) in the context of association test statistics from genome-wide large-scale association studies, we divide the human genome into consecutive blocks of 1 cM length, group the association test statistics within these blocks into clusters and use the estimated LD matrices as working-correlation matrices. For most association studies, reference populations from reference panels such as the 1000 Genomes Project will be available that mirror the LD structure of the study/target population reasonably well. In contrast to the standard application of the GEE approach, the clusters of observations/association test statistics are here generally not statistically independent, as association test statistics in adjacent clusters will be correlated due to LD. However, since the length of the blocks of 1 cM is chosen sufficiently large, we can assume that only nearby clusters are correlated. In Appendix B of the supplementary materials available at *Biostatistics* online, we show how the original GEE assumption of independent clusters can be relaxed in such a way, i.e. the sandwich-variance estimator can be extended to the scenario of sparsely correlated clusters and how the classical asymptotic results can be reestablished. Now, we describe the GEE objects to estimate the parameters in the mean model for the association test statistics. The following description and the derivations in the supplementary materials available at *Biostatistics* online are strongly related to Wang (2011) as well as Xie and Yang (2003). As equations (2.1) and (2.2) imply the setting of gamma-distributed test statistics $ \chi^{2}_l$, we use the exponential family distribution, including the gamma distribution, using the general notation as in Xie and Yang (2003). As described above, we arrange the human genome respectively the association test statistics $ \chi^{2}_l$, $ l=1,\dots,n_{\rm SNPs}$, into 1 cM clusters. The input is therefore described in the following by $ y_i=(y_{i1}, \dots,y_{im_i } )^T,i=1,\dots,n$, where $ n$ is the number of clusters with bounded size $ m_i$. In addition, we have covariates $ x_i=(x_{i1},\dots,x_{im_i } )^T$, where $ x_{ij}$ is a $ p_n \times 1$ vector. $ p_n$ denotes the number of parameters of interest. Although the number of parameters $ p_n=2$ and the covariate design (2.1) is fixed in the application described in this work, we allow general covariate data and $ p_n$ to grow with the number of clusters for future applications (see Appendix B of the supplementary materials available at *Biostatistics* online). We denote the true, unknown parameter by $ \beta_{n0}$. The mean and variance are described by $ \mathbf{E}[\,y_{ij} | x_{ij},\beta_n, \phi]=a^{'} (\theta_{ij} )$ and $ \sigma^{2}_{ij}=\text{Var}(y_{ij} | x_{ij},\beta_n,\phi)=\phi a^{''}(\theta_{ij}) $, where $ \theta_{ij}=u(x^{T}_{ij} \beta_n )$ and $ u$ is the injective link function (Xie and Yang, 2003). We use the notation $ \mu_i (\beta_n )=(\mu_{i1} (\beta_n )\dots,\mu_{im_i} (\beta_n ) )^{T}, A_i (\beta_n)=diag(\sigma^{2}_{i1} (\beta_n ),\dots,\sigma^{2}_{im_i} (\beta_n )) $ and $ \Delta_i (\beta_n )=diag(u'(x^{T}_{i1} \beta_n),\dots,u'(x^{T}_{im_i} \beta_n ))$ as in Xie and Yang (2003). The specification of the functions $ a$ and $ u$ for our setting follows from the gamma distribution representation. We also define the GEE objects $ D_i (\beta_n )=A_i (\beta_n ) \Delta_i (\beta_n ) X_i, V_i (\beta_n )=A^{{1}/{2}}_i (\beta_n ) R_i A^{1/2}_i (\beta_n )$ and $ H_n (\beta_n )=\sum\limits_{i=1}^n D^{T}_i (\beta_n ) V^{-1}_i (\beta_n ) D_i (\beta_n)$. $ R_i$ denotes the working-correlation matrix for cluster $ i$. The entries of the working-correlation matrices are defined by the corresponding estimated LD measure $ r^{2}$. This is the extension for the correlation of squared test statistics from the well-established correlation approximation of normally distributed association z-scores equal to the LD measure $ r$. With these objects, we can set up the GEEs

(2.8) \begin{equation*} g_n (\beta_n )= \sum\limits_{i=1}^{n} D^{T}_i ( \beta_n ) V^{-1}_i (\beta_n )(y_i-\mu_i (\beta_n ) )=0. \end{equation*}

Under the assumption of sparsely correlated clusters and reasonable technical conditions (Appendix B of the supplementary materials available at *Biostatistics* online), we can reestablish the following results for the asymptotic existence of the estimator $ \hat{\beta}_n$, which are oriented at previous work (Wang, 2011). The proofs are sketched in Appendix B of the supplementary materials available at *Biostatistics* online. The two key points in the proofs of Propositions 3 and 5 are the utilization of a central limit theorem for weakly dependent data and the modification of the sandwich-variance estimator to account for the specified dependence structure of the clusters.

Proposition 2 There exists a root $ \hat{\beta}_n$ of $ g_n ( \beta_n )=0$ satisfying $ ||\hat{\beta}_n-\beta_{n0} ||=O_p \bigg(\frac{\sqrt{p_n}}{\sqrt{n}}\bigg)$.

Since the clusters are only sparsely correlated, we still observe the asymptotic normality of $ g_n ( \beta_{n0}) $.

Proposition 3 For $ \alpha_n \in \mathrm{R}^{p_n}$ with $ ||\alpha_n ||=1$, we have $ \alpha^{T}_n M^{-\frac{1}{2}}_n (\beta_{n0} ) g_n (\beta_{n0} ) \to N(0,1)$, as $ n \to \infty$, in distribution, where $ M_n(\beta_{n0})=\text{Cov}(g_n(\beta_{n0}))$.

With these results, we can establish the asymptotic distribution of the estimator $ \hat{\beta}_n$.

Proposition 4 $ \alpha^{T}_n M^{-\frac{1}{2}}_n (\beta_{n0} ) H_n (\beta_{n0} )(\hat{\beta}_n-\beta_{n0}) \to N(0,1)$, as $ n \to \infty$, in distribution.

Finally, we analyze the features of the estimators variance and the corresponding hypothesis testing. Therefore, define the covariance matrix $ \Sigma_n=H^{-1}_n (\beta_{n0} ) M_n (\beta_{n0} ) H^{-1}_n (\beta_{n0} )$.

Proposition 5 For an $ l \times p_n$ matrix $ C_n$ such that $ G=C_n C^{T}_n$ with $ G$ positive definite, we have $ C_n \hat{\Sigma}_n C^{T}_n-C_n \Sigma_n C^{T}_n= o_p (n^{-1} )$, where $ \hat{\Sigma}_n =H^{-1}_n (\hat{\beta}_n) \hat{M}_n (\hat{\beta}_n) H^{-1}_n (\hat{\beta}_n)$ and $ \hat{M}_n (\beta)=\sum\limits_{i=1}^{n} g_{ni} (\beta) g^{T}_{ni} (\beta) + \sum\limits_{i \ne j \text{ correlated}} g_{ni} (\beta) g^{T}_{nj} (\beta)$. $ \hat{\Sigma}_n$ describes the sandwich-variance estimator.

If we are interested to test hypotheses of the form $ H_0: \beta_{n0b}=0$ vs. $ H_1: \beta_{n0b} \ne 0$ for $ b=1,\dots,p_n$, we can use the immediate consequence (Wang, 2011):

Corollary 2.1 Under $ H_0$, $ W_{nb}=\frac{\hat{\beta}^{2}_{nb}}{\hat{\Sigma}_{nbb}} \to \chi^{2}(1)$, as $ n \to \infty$, in distribution, where $ \chi^{2}(1)$ denotes the chi-squared distribution with one degree of freedom.

To solve the GEEs, we implemented a C/C++ tool to extract the working-correlation matrices from the 1000 Genomes data and solve the GEEs via a Newton-Raphson algorithm. For the covariates, we used the pre-computed LD Scores for the European sample of 1000 Genomes (1000 Genomes Project Consortium *and others*, 2012).

## 3. Results

In the following section, we present the results of a simulation study and the analysis of real data examples.

### 3.1. Simulation study

We demonstrate the features of the proposed method PolyGEE by simulation studies in which we mimicked the summary statistics from a large-scale association study. A crucial property of the simulation setting is a realistic LD structure of the simulated test statistics. We utilized the LD structure of chromosome 2 in the European sample of the 1000 Genomes Project (1000 Genomes Project Consortium *and others*, 2012). We randomly selected 62 500 common variants for which pre-calculated LD Scores are available from the LD Score regression project (Bulik-Sullivan *and others*, 2015b). The corresponding distances between the genetic positions of these variants, calculated by the genetic map of the 1000 Genomes project, ranged from approximately 0–275 cM. Due to computational restrains for large matrices, we partitioned the chromosome in 11 regions of about 25 cM length. For each region, we estimated the LD matrix between the variants from the phased 1000 Genomes project data set. Between variants with more than 1 cM distance, LD estimates were truncated to zero. We used the resulting LD matrix as the correlation matrix of normally distributed, mean zero *z*-scores with variance

\begin{equation*}
\beta_1+l_l \beta_2,
\end{equation*}

where $ l_l$ is the LD Score of the corresponding variant $ l$. To achieve the setting of genome-wide data, we repeated 13 independent draws of these variants and obtained 812 500 *z*-scores, respectively, squared test statistics. In conclusion, these squared test statistics follow our general marginal model equations (2.1) and (2.2) and the assumed band structure of LD/correlation. To investigate multiple scenarios, we assigned different combinations of reasonable values to the two parameters $ \beta_1$ and $ \beta_2$, similar to estimated results from real data. For PolyGEE, we split each region into 1 cM blocks, which resulted in about 25 blocks for each region. For each block, we extracted the correlation structure between the test statistics from the corresponding LD matrix. These LD matrices for the 1 cM blocks were used as the working-correlation matrices. We first ran the PolyGEE analysis with these original LD matrices, mimicking the scenario where the LD from the reference panel coincides with the LD structure in the data set. In a second set of simulations, we ran the analysis with a scaled version of these matrices, where we shrinked the LD matrices towards the identity matrix with a factor of 64%. This set of simulations mimics the scenario where LD matrices substantially deviate from the true correlation structure. In order to compare the performance of PolyGEE, we implemented an explicit weighted linear regression estimator of the model of LD Score regression (Bulik-Sullivan *and others*, 2015b) (2.4 and 2.5). For the corresponding heteroscedasticity weights, we applied the true, in practice unknown, values for the parameters. The overcounting part of the weights was correctly specified. The LDSC software for LD Score regression uses a two-step delete-block jackknife procedure (200 consecutive blocks per default) for the estimation of parameters and standard errors (Bulik-Sullivan *and others*, 2015b). As described, we used an explicit estimator instead and estimated the empirical standard error over independent replications. This also avoided the difficult choice of the number of blocks for the jackknife in LDSC for our setting, which clearly influences the results in practice. Table 1 contains the empirical and estimated standard errors of the PolyGEE estimates for 8 different parameter value combinations. The empirical standard errors were computed over 1000 replications, the estimated standard errors were calculated by the sandwich-variance formula (see Proposition 5) and averaged over the replications. The simulation results confirm that the variance estimator correctly estimates the standard errors of our method PolyGEE, in the setting of full correlation information as well as in the scenario of the shrinked information. Table 2 provides the relative efficiency of the LD Score regression estimator in comparison with PolyGEE. We observed that the LD Score regression estimation variance is up to more than factor 2 larger than the estimated variances of our PolyGEE approach, if the LD working-correlation matrices are correctly specified. The relative efficiencies of LD Score regression compared to PolyGEE range between 47% and 67%. If the LD working-correlation matrices are strongly shrinked towards the identity matrix, the gain of efficiency is smaller, but still substantial. The relative efficiencies along both parameters in this shrinked scenario range between 73% and 86%, with median and mean of 79%.

**Table 1.** Empirical standard errors (emp. s.e.) over 1000 replications and the estimated standard errors (est. s.e.) for PolyGEE are listed for the full and shrinked information scenario. Standard errors for the first parameter in $ 10^{-3}$ and $ 10^{-5}$ for the second

PolyGEE standard error estimation								
	98%				64%			
	emp. s.e.		est. s.e.		emp. s.e.		est. s.e.	
$ (\beta_1,\beta_2)$	$ \beta_1$	$ \beta_2$	$ \beta_1$	$ \beta_2$	$ \beta_1$	$ \beta_2$	$ \beta_1$	$ \beta_2$
$ (1.00,0.0000)$	5.11	4.11	5.08	4.06	5.67	5.25	5.62	5.21
$ (1.00,0.0001)$	4.98	4.22	5.14	4.23	5.50	5.33	5.68	5.36
$ (1.00,0.0005)$	5.37	4.95	5.35	4.83	5.95	6.06	5.89	5.88
$ (1.00,0.0010)$	5.67	5.58	5.60	5.50	6.20	6.54	6.12	6.48
$ (1.02,0.0000)$	5.20	4.07	5.18	4.16	5.76	5.25	5.74	5.33
$ (1.02,0.0001)$	5.25	4.31	5.23	4.31	5.77	5.38	5.80	5.46
$ (1.02,0.0005)$	5.50	4.82	5.45	4.91	6.02	5.87	6.00	5.99
$ (1.02,0.0010)$	5.53	5.59	5.60	5.50	6.13	6.61	6.13	6.48

**Table 2.** Relative efficiency of the LD Score regression estimator in comparison with PolyGEE estimator, based on 1000 replications. The efficiencies are listed for the full and shrinked information scenario

	98%		64%	
$ (\beta_1,\beta_2)$	$ \beta_1 (\%)$	$ \beta_2 (\%)$	$ \beta_1 (\%)$	$ \beta_2(\%)$
(1.00,0.0000)	61.2	49.0	75.4	80.0
(1.00,0.0001)	61.9	53.1	75.5	84.7
(1.00,0.0005)	63.3	55.7	77.7	83.5
(1.00,0.0010)	66.6	61.6	79.6	84.6
(1.02,0.0000)	59.9	47.1	73.5	78.4
(1.02,0.0001)	63.9	52.6	77.4	81.8
(1.02,0.0005)	63.2	55.5	75.7	82.4
(1.02,0.0010)	61.5	61.1	75.6	85.5

### 3.2. Application to real data

In this section, we compare the performance of PolyGEE with LD Score regression based on the association results for public available summary statistics.

#### 3.2.1. Population-based association data.

We applied both methods, PolyGEE and LD Score regression, to the summary association statistics of three traits from the PGC. The PGC provides the results of large-scale meta-analyses for bipolar disorder (BIP), schizophrenia (SCZ), major depressive disorder (MDD), attention deficit disorder (ADHD), and autism spectrum disorder (ASD). We analyzed the data from the PGC-Cross-Disorder Group for SCZ (Cross-Disorder Group of the Psychiatric Genomics Consortium *and others*, 2013) and the meta-analysis results for BIP (Psychiatric GWAS Consortium Bipolar Disorder Working Group *and others*, 2011) and MDD (Ripke *and others*, 2013). The samples for these diseases are unrelated case-control cohorts.

We filtered the summary statistics with the LDSC software (Bulik-Sullivan *and others*, 2015b) using the default parameters. This procedure includes removing ambiguous SNPs and variants with an imputation info-score $ <$ 0.9, if the info-score is available. If no imputation-info score is available it is suggested to restrict the input to the set of HapMap3 (International HapMap 3 Consortium *and others*, 2010) SNPs, since these SNPs are usually well-imputed. We used both filter options for the PGC studies. LD Score regression was performed with LDSC, based on the pre-calculated LD Scores for the European Sample of the 1000 Genomes project. For PolyGEE, we took the same input data set as for LD Score regression, but additionally excluded SNPs in perfect LD and variants with very low p-values ($ <$ e-16) (if exist). This excludes model outliers and avoids the construction of degenerated correlation matrices. Table 3 provides the estimated parameters and the corresponding number of variants included in this analysis. The polygenic term corresponds to an estimate of the heritability on the observed scale, using the number of SNPs $ M$ for common SNPs (see above) provided by LDSC and the reported sample size from the corresponding publication. Therefore, the polygenic term quantifies $ h^2 \frac{c^2 n_{\rm cases} n_{\rm controls}}{N^2 (1-K)^2 }$. Since we compare the performance of estimators in terms of accuracy, we renounce to transform the heritability to the liability scale, since this is a constant factor transformation of the estimate. The estimated standard errors of the LD Score regression estimates were calculated by LDSC using the default settings. For PolyGEE, the estimated standard errors were calculated by our sandwich-variance estimator (Proposition 5). We observe that the PolyGEE estimates are more accurate and that the variance of the parameter estimates for the LD Score regression is around 50% higher.

**Table 3.** Results for the population-based meta-analyses for BIP, SCZ, and MDD of the PGC. We provide the estimates for LD Score regression and PolyGEE. Standard errors depicted in brackets

Disorder	LDSC			PolyGEE		
	#SNPs	$ 1+Na$	Polygenic term	#SNPs	$ 1+Na$	Polygenic term
BIP	803 530	1.0495 (0.0105)	0.3046 (0.0379)	667 734	1.0448 (0.0083)	0.2953 (0.0312)
SCZ	840 450	1.0418 (0.0117)	0.4190 (0.0455)	761 854	1,0428 (0.0091)	0.3709 (0.0380)
MDD	891 172	1.0241 (0.0079)	0.1130 (0.0265)	812 873	1,0181 (0.0065)	0.1093 (0.0223)

#### 3.2.2. Family-based association data.

We also applied PolyGEE and LD Score regression to the summary statistics from the Autism Genome Project (AGP) Consortium association study for autism spectrum disorder (ASD) (Anney *and others*, 2012). The analysis was based on 2,359 affected offspring of European ancestry. Quality control of the input data was performed as for the population-based association studies. It is important to note again that, despite that LD Score regression was developed for population-based association statistics, the derivation of equation (2.7) implies the possibility to also analyze family-based association by the underlying estimation framework of LD Score regression. Since the output options of the LDSC software assume population-based association data, we had to modify the results and the output according to equation (2.7). Table 4 contains the results of the analysis with PolyGEE and LDSC. The polygenic term describes $ hc^2$ ($  c^2$ as defined in equation (2.3)), assuming 2,359 trios as an accurate approximation of the family structure in the sample (Anney *and others*, 2012). The findings in terms of efficiency gains are substantial and of similar magnitude as for the population-based studies.

**Table 4.** Results for the family-based association study for ASD. We provide the estimates for LD Score regression and PolyGEE. Standard errors depicted in brackets

Disorder	LDSC			PolyGEE		
	#SNPs	$ 1+Na$	Polygenic term	#SNPs	$ 1+Na$	Polygenic term
ASD	806 205	1.0396 (0.0073)	0.5754 (0.4052)	701 164	1.0387 (0.0056)	0.5750 (0.3251)

## 4. Discussion

At a genome-wide level, we derived a general marginal model for single-variant association test statistics of complex diseases. We proposed the PolyGEE methodology to estimate the amount of polygenic effects and confounding biases from the association test statistics of large-scale association studies using this model. We showed via simulation studies and the application to real data that our approach is substantially more efficient than the existing LD Score regression framework, i.e. the estimates are more precise. The increased efficiency of our approach is achieved by incorporating detailed local LD information from the external reference panels, e.g. 1000 Genomes project, into the estimation step. However, our GEE approach does not require that the reference panel and the study data are exact matches in terms of the LD structure. The approach is robust against deviations of the sample LD structure from the reference panel and can compute asymptotic valid standard errors. Our theoretical derivations and assumptions lead to general valid results for the estimation framework. For further research, this makes it possible to extend the mean model to incorporate more components or estimate the genetic correlation between two traits.

## Supplementary Material

Supplementary Data
