# Supplementary material for: PolyGEE: a generalized estimating equation approach to the efficient and robust estimation of polygenic effects in large-scale association studies
Source: Biostatistics. 2017 Aug 31;19(3):295–306. doi: 10.1093/biostatistics/kxx040 (PMC5991211; doi:10.1093/biostatistics/kxx040)
Supplement: Supplementary Data [file kxx040_supp.pdf]

*Biostatistics* (2017), **0**, 0, *pp.* 1–20
doi:10.1093/biostatistics/supplement

# PolyGEE: A generalized estimating equation approach to the efficient and robust estimation of polygenic effects in large-scale association studies

JULIAN HECKER*[,1,3], DMITRY PROKOPENKO[2],

CHRISTOPH LANGE[1,2], HEIDE LOEHLEIN FIER[1,3]

**Supplementary Materials**

*1.)Department of Biostatistics, Harvard T.H. Chan School of Public Health, 655 Huntington Avenue, Boston, MA 02115, USA*
*2.)Channing Division of Network Medicine, Brigham and Women's Hospital, 181 Longwood Avenue, Boston, MA 02115, USA*

*3.)Department of Genomic Mathematics, University of Bonn, Sigmund-Freud-Strasse 25, 53127 Bonn, Germany*

jhecker@hsph.harvard.edu

## Appendix A: Approximate power of family-based association studies

In the following section, we analyze the behavior of family-based association statistics for a broad class of family settings.

### *A.1 Proof of Proposition 1*

We will show how to derive Proposition 1. First, we start with a general probabilistic result.

Sums of multinomial distributed components:

Assume we have two different probability measures, denoted by $H = 0, A$. We consider a multino-

*To whom correspondence should be addressed.

© The Author 2017. Published by Oxford University Press. All rights reserved. For permissions, please e-mail: journals.permissions@oup.com

mial distribution, with respect to two measures $s^H$ with parameterization $s^H \sim \text{Multinomial}(N, s_1^H, \ldots, s_k^H)$. Furthermore, we have $k$ sub distributions with finite expectations $\mu_i^H$ and finite variances $\sigma_i^{2H}$ . Now, we construct a random variable $Z_N$ as follows:

$$Z_N = \sum_{i=1}^{k} \sum_{j=1}^{S_i^A} X_{ij}^A,$$

where $S^A = (S_1^A, \ldots, S_k^A)$ is a realization of $s^A$ and $X_{ij}^A$ are independent realizations of the corresponding sub distribution. By standard arguments, we conclude that, under the measure $A$,

$$\frac{Z_N - \mathrm{E}_A[Z_N]}{\sqrt{\mathrm{Var}_A[Z_N]}} \to N(0,1) \text{ as } N \to \infty \text{ in distribution,}$$

with

$$\mathrm{E}_A[Z_N] = N \sum_{i=1}^{k} \mu_i^A s_i^A$$

and, by the law of total variance,

$$\mathrm{Var}_A[Z_N] = N \Bigg[ \sum_{i=1}^{k} s_i^A (\mu_i^A - \sum_{j=1}^{k} s_j^A \mu_j^A)^2 + \sum_{j=1}^{k} s_i^A \sigma_i^{2A} \Bigg].$$

If we now consider the random variable $T_N$, constructed as

$$T_N = \frac{\sum_{i=1}^{k} \sum_{j=1}^{S_i^A} [X_{ij}^A - \mu_i^0]}{\sqrt{\sum_{i=1}^{k} S_i^A \sigma_i^{20}}},$$

we conclude that, under $A$, $T_N$ is asymptotically normally distributed with mean

$$\mu_N = \sqrt{N} \frac{\sum_{i=1}^{k} s_i^A [\mu_i^A - \mu_i^0]}{\sqrt{\sum_{i=1}^{k} s_i^A \sigma_i^{20}}}$$

and variance

$$\sigma_N^2 = \frac{\sum_{i=1}^{k} s_i^A [\mu_i^A - \mu_i^0 - \sum_{j=1}^{k} s_i^A (\mu_i^A - \mu_i^0)]^2 + \sum_{i=1}^{k} s_i^A \sigma_i^{2A}}{\sum_{i=1}^{k} s_i^A \sigma_i^{20}},$$

since

$$\frac{1}{\sqrt{N}}\sqrt{\sum_{i=1}^{k} S_i^A \sigma_i^{20}} \to \sqrt{\sum_{i=1}^{k} s_i^A \sigma_i^{20}} \text{ as } N \to \infty$$

in probability.

Application to FBAT

The test statistic of FBAT (Lange and Laird (2002)) can be described as $T_N^2$ . To see this, we fix the ascertainment condition, for example *affected trios from random families* and interpret the random variable $s^H$ as the number of families of a specific type (in terms of the sufficient statistic). We restrict us to the scenario where parental genotypes are available. In this setting, the sufficient statistic equals the parental genotypes. The $k$ sub distributions and their means and variances correspond to the conditional marker distributions in offspring and the specific family type, under the null measure $H = 0$ or the alternative $H = A$. This description equals the derivations for the software PBAT (Lange and Laird (2002)). As established in the existing literature, we describe the disease model by a multiplicative relative risk model with relative risk $\lambda$ and prevalence $K$ (Yang *and others* (2011), Peyrot *and others* (2016)). To derive the approximation stated in Proposition 1, we analyze the expression

$$\mathrm{E}_A[T_N^2] = \sigma_N^2 + \mu_N^2.$$

We use the approximation $\sigma_N^2 = 1$, which is very accurate in the usual scenarios. The main driver of $\mathrm{E}_A[T_N^2]$ is $\mu_N^2$. Since we parametrized the distributions with the relative risk $\lambda$, we can identify the measure $H = A$ with $\lambda \neq 1$ and $H = 0$ with $\lambda = 1$. In addition, we can derivate the corresponding expectations. Thus, we compute a second order Taylor approximation of $\mu_N^2$ with respect to $\lambda$ at $\lambda = 1$. We have

$$\mu_N^2 = N \frac{\left[\sum_{i=1}^{k} s_i^A [\mu_i^A - \mu_i^0]\right]^2}{\sum_{i=1}^{k} s_i^A \sigma_i^{20}}.$$

Thus, the 0-th and 1-th term vanish and we obtain

$$\mathrm{E}_A[T_N^2] \approx 1 + \frac{1}{2} N \frac{2\left[\sum_{i=1}^{k} s_i^0 (\mu_i^A)'_{|\lambda=1}\right]^2}{\sum_{i=1}^{k} s_i^0 \sigma_i^{20}} (\lambda - 1)^2 (A1)$$

Evaluation of the expressions for given ascertainment condition

Now, we evaluate the expressions in A.1 for a given ascertainment condition. Assume that families with $n_a$ affected and $n_u$ unaffected offspring are selected. The phenotypes of the parents are arbitrary. We assume independence of transmissions and that the probability of disease status only depends on the genotype of the corresponding offspring, similar to Eq. (9) in Lange and Laird (2002). Let denote the offset parameter. Thus, the two possible phenotype codings are $1 - z$ for affected and $z$ for unaffected offspring. Under the independence assumption, the mean of the conditional marker distribution within a family can be treated independently (compare again with Eq. (9) in Lange and Laird (2002). The same holds for the variance of the conditional marker distribution. This implies

$$\sum_{i=1}^{k} s_i^0 \sigma_i^{20} = ((1-z)^2 n_a + z^2 n_u) p(1-p).$$

The first factor comes from the phenotype coding. The $p(1-p)$ is the result of the observation that the variance of the conditional marker distribution is 1/2 if both parents are heterozygotes, 1/4 if only one parent is a heterozygote and 0 else. The probability for a parent to be heterozygote is $2p(1-p)$. Finally, we have to evaluate $\sum_{i=1}^{k} s_i^0 (\mu_i^A)'_{|\lambda=1}$.

Introduce the notation $\mathrm{E}_0[X|s_i]$ for the genotype marker expectation (additive coding) under the null, conditioned on the i-th family type. Recall from Eq. (9) in Lange and Laird (2002):

$$p_A(x|y, s_i) = \frac{p_A(y|x) p(x|s_i)}{\sum_{x'} p_A(y|x') p(x'|s_i)},$$

same notational context as for the conditional expectation. Relaxing the assumption that the tested marker equals the true DSL, denoted by $D$ and with MAF $f$, and plugging in the expres-

sions of the disease model, we obtain by simple, but burdensome calculations:

$$\sum_{i=1}^{k} s_i^0 (\mu_i^A)'_{|\lambda=1} = \left( (1-z) n_a \frac{K}{1-K} z n_u \right) \sum_{i=1}^{k} s_i^0 (\mathrm{E}_0[XD|s_i] - \mathrm{E}_0[D|s_i]\mathrm{E}_0[X|s_i]).$$

We have

$$\sum_{i=1}^{k} s_i^0 (\mathrm{E}_0[XD|s_i] - \mathrm{E}_0[D|s_i]\mathrm{E}_0[X|s_i]) = \mathrm{E}_0[\mathrm{Cov}_0(X, D|s_i)]$$

and

$$\mathrm{E}_0[\mathrm{Cov}_0(X, D|s_i)] = 2D - 2p2f - D + 2p2f = D$$

with $D = r\sqrt{p(1-p)f(1-f)}$ where $r$ is the LD measure between both loci.

Overall, this results in

$$\mathrm{E}_A[T_N^2] \approx 1 + r^2 \frac{1}{2} N \frac{\left[ (1-z) n_a + \frac{K}{1-K} z n_u \right]^2}{(1-z)^2 n_a + z^2 n_u} 2p(1-p)(\lambda - 1)^2,$$

resulting in Proposition 1 using Eq. (3). This proves the approximation stated in Proposition 1.

### *A.2 Mixed types of families*

If $N_1$ families are selected with respect to a first ascertainment condition and $N_2$ families with respect to a different ascertainment condition, the extension is straightforward. One can construct a new, combined multinomial distribution with sample size $N = N_1 + N_2$ and rescaled parameters. The result for the approximation is just the appropriate rescaled version of the single results.

## Appendix B: GEE results

We derive the results in a general setting, where the distribution of the responses is modeled by an exponential family distribution.

The derivations and results are strongly related and connected to the work in Wang (2011) and present slight modifications to our setting. We extend and modify the respective results to our setting with the help of results in Xie and Yang (2003) and Lumley and Mayer Hamblett (2003). The most important difference is the relaxation to sparsely correlated clusters instead of independent clusters.

First, we describe the underlying objects and notation.

### *B.1 Setting*

Denote by $G_n$ the negative derivative of $g_n(\beta)$:

$$G_n(\beta_n) = -\frac{\partial g_n(\beta_n)}{\partial \beta_n}.$$

Introduce the notation

$$\varepsilon_i(\beta_n) = A_i^{-\frac{1}{2}}(\beta_n)(y_i - \mu_i(\beta_n)).$$

with corresponding components $\varepsilon_{ij}(\beta_n)$, $j = 1, \dots, m_i$.

### *B.2 Assumptions*

**Assumption 0.1.** *1)* $sup_{i,j} ||X_{ij}|| = O(\sqrt{p_n})$
*2)* $0 < C_1 < \lambda_{min}\left(\frac{1}{n}\sum_{i=1}^{n} X_i^\intercal X_i\right) \leqslant \lambda_{max}\left(\frac{1}{n}\sum_{i=1}^{n} X_i^\intercal X_i\right) < C_2 < \infty$, *for all* $n$.
*3)* $R_i$ *positive definite for all* $i = 1, \dots, n$.
*4)* $\mathrm{E}[||A^{-\frac{1}{2}}(\beta_n)(y_i - \mu_i(\beta_n))||^4] < \infty$ *in a neighborhood of* $\beta_{n0}$.
*5) The true correlation matrix for all responses* $y_{ij}$, $i = 1, \dots, n$, $j = 1, \dots, m_i$ *is positive definite. The submatrix* $\bar{R}_i$ *for cluster* $i$ *is positive definite for all* $i = 1, \dots, n$.
*6) The entries of* $A_i(\beta_n)$, $\Delta_i(\beta_n)$, $G_i^{[1]}(\beta_n)$, $G_i^{[2]}(\beta_n)$ *(defined below) and derivatives of the link function* $u_i(\beta_n)$ *are uniformly bounded in a neighborhood of* $\beta_{n0}$.

*7)* $\frac{p_n^4}{n} \to 0$ *as* $n \to \infty$.

*8) The number of neighbor clusters correlated with cluster* $i$ *is uniformly bounded by a small constant (in our scenario we can assume this constant to be 1).*

### *B.3 Proofs*

*Proof Proposition 2.* Following Wang (2011), we only have to evaluate the sign of $(\beta_n - \beta_{n0})^\intercal g_n(\beta_n)$ on

$$\left\{\beta_n : ||\beta_n - \beta_{n0}|| = \Delta\sqrt{\frac{p_n}{n}}\right\}, \quad \Delta > 0.$$

Write

$$(\beta_n - \beta_{n0})^\intercal g_n(\beta_n) = (\beta_n - \beta_{n0})^\intercal g_n(\beta_{n0}) - (\beta_n - \beta_{n0})^\intercal G_n(\beta_n^*)(\beta_n - \beta_{n0}) =: I_1 + I_2,$$

where $\beta_n^*$ lies between $\beta_n$ and $\beta_{n0}$.

We can decompose $I_2$ in

$$I_2 = -(\beta_n - \beta_{n0})^\intercal H_n(\beta_{n0})(\beta_n - \beta_{n0})$$

$$-(\beta_n - \beta_{n0})^\intercal [H_n(\beta_n^*) - H_n(\beta_{n0})](\beta_n - \beta_{n0})$$

$$-(\beta_n - \beta_{n0})^\intercal [G_n(\beta_n^*) - H_n(\beta_n^*)](\beta_n - \beta_{n0}) =: I_{21} + I_{22} + I_{23}.$$

Direct computation gives

$$I_{21} \leqslant -C\Delta^2 p_n.$$

In addition, we have

$$|I_1| \leqslant ||\beta_n - \beta_{n0}||\ ||g_n(\beta_{n0})|| = \Delta\sqrt{\frac{p_n}{n}}||g_n(\beta_{n0})||.$$

From the Lemma 0.2, 0.3 and 0.4, we conclude

$$I_{22} = \Delta^2 o_p(p_n),\ I_{23} = \Delta^2 o_p(p_n) \text{ and } |I_1| = \Delta O_p(p_n),$$

Therefore, $(\beta_n - \beta_{n0})^\intercal g_n(\beta_n)$ on $\{\beta_n : ||\beta_n - \beta_{n0}|| = \Delta\sqrt{\frac{p_n}{n}}\}$, $\Delta > 0$, is asymptotically dominated in probability by $I_1 + I_{21}$, which is negative for $\Delta$ large enough.

□

We need the following helpful results.

**Lemma 0.2.**

$$\sup_{||\beta_n-\beta_{n0}||\leqslant\Delta\sqrt{\frac{p_n}{n}}}\sup_{||b_n||=1}[b_n^\intercal[H_n(\beta_n)-H_n(\beta_{n0})]b_n]=O_p(\sqrt{n}p_n).$$

*Proof.* By definition,

$$b_n^\intercal[H_n(\beta_n)-H_n(\beta_{n0})]b_n=$$

$$\leqslant\left|\sum_{i=1}^n b_n^\intercal X_i^\intercal[\Delta_i(\beta_n)A_i^{\frac{1}{2}}(\beta_n)R_i^{-1}A_i^{\frac{1}{2}}(\beta_n)\Delta_i(\beta_n)-\Delta_i(\beta_n)A_i^{\frac{1}{2}}(\beta_n)R_i^{-1}A_i^{\frac{1}{2}}(\beta_{n0})\Delta_i(\beta_{n0})]X_ib_n\right|$$

$$+\left|\sum_{i=1}^n b_n^\intercal X_i^\intercal[\Delta_i(\beta_n)A_i^{\frac{1}{2}}(\beta_n)R_i^{-1}A_i^{\frac{1}{2}}(\beta_{n0})\Delta_i(\beta_{n0})-\Delta_i(\beta_{n0})A_i^{\frac{1}{2}}(\beta_{n0})R_i^{-1}A_i^{\frac{1}{2}}(\beta_{n0})\Delta_i(\beta_{n0})]X_ib_n\right|=:I_1+I_2$$

Decomposition yields

$$I_1\leqslant\sum_{i=1}^n\left|\left|b_n^\intercal X_i^\intercal\Delta_i(\beta_n)A_i^{\frac{1}{2}}(\beta_n)R_i^{-\frac{1}{2}}\right|\right|\,\left|\left|R_i^{-\frac{1}{2}}[A_i^{\frac{1}{2}}(\beta_n)\Delta_i(\beta_n)-A_i^{\frac{1}{2}}(\beta_{n0})\Delta_i(\beta_{n0})]X_ib_n\right|\right|.$$

We have

$$||b_n^\intercal X_i^\intercal\Delta_i(\beta_n)A_i^{\frac{1}{2}}(\beta_n)R_i^{-\frac{1}{2}}||\leqslant\max_i\lambda_{\max}^{\frac{1}{2}}(R_i^{-1})\max_i\lambda_{\max}^{\frac{1}{2}}(A_i(\beta_n))\max_i\lambda_{\max}^{\frac{1}{2}}(\Delta_i^2(\beta_n))||X_ib_n||.$$

Similar,

$$||R_i^{-\frac{1}{2}}[A_i^{\frac{1}{2}}(\beta_n)\Delta_i(\beta_n)-A_i^{\frac{1}{2}}(\beta_{n0})\Delta_i(\beta_{n0})]X_ib_n||$$

$$\leqslant\max_i\lambda_{\max}^{\frac{1}{2}}(R_i^{-1})\max_i\lambda_{\max}(A_i^{\frac{1}{2}}(\beta_{n0})\Delta_i(\beta_{n0})-A_i^{\frac{1}{2}}(\beta_n)\Delta_i(\beta_n))||X_ib_n||.$$

Thus,

$$I_1\leqslant C\max_{i,j}||X_{ij}||\,||\beta_n-\beta_{n0}||\lambda_{\max}\left(\sum_{i=1}^n X_i^\intercal X_i\right)$$

and therefore,

$$\sup_{||\beta_n-\beta_{n0}||\leqslant\Delta\sqrt{\frac{p_n}{n}}}\sup_{||b_n||=1}I_1=O_p(\sqrt{n}p_n).$$

$I_2$ can be treated in the same way, completing the statement. □

**Lemma 0.3.**

$$\sup_{||\beta_n-\beta_{n0}||\leqslant\Delta\sqrt{\frac{p_n}{n}}}\ \sup_{||b_n||=1}[b_n^\intercal[G_n(\beta_n)-H_n(\beta_n)]b_n]=O_p(\sqrt{n}p_n).$$

*Proof.* We use the decomposition of $G_n(\beta_n)$ as in Xie and Yang (2003). This decomposition is given by

$$G_n(\beta_n)=H_n(\beta_n)+B_n^{[1]}(\beta_n)+B_n^{[2]}(\beta_n)+E_n^{[1]}(\beta_n)+E_n^{[2]}(\beta_n).$$

Furthermore, let $\mu_i^0:=\mu_i(\beta_{n0})$.

The diagonal matrices $G_n^{[1]}(\beta_n)$ and $G_n^{[2]}(\beta_n)$ are defined in Xie and Yang (2003) and bounded by Assumption A6. We can estimate

$$|b_n^\intercal[B_n^{[2]}(\beta_n)]b_n|$$

$$\leqslant C\sup_{i,j}||X_{ij}||\sum_{i=1}^{n}\left(b_n^\intercal X_i^\intercal X_i b_n\right)||\beta_n-\beta_{n0}||.$$

Therefore,

$$\sup_{||\beta_n-\beta_{n0}||\leqslant\Delta\sqrt{\frac{p_n}{n}}}\ \sup_{||b_n||=1}[b_n^\intercal[B_n^{[2]}(\beta_n)]b_n]=O(\sqrt{p_n})O_p\left(\sqrt{\frac{p_n}{n}}\right)O(n)=O_p(\sqrt{n}p_n).$$

Similar,

$$\sup_{||\beta_n-\beta_{n0}||\leqslant\Delta\sqrt{\frac{p_n}{n}}}\ \sup_{||b_n||=1}[b_n^\intercal[B_n^{[1]}(\beta_n)]b_n]=O(\sqrt{p_n})O_p\left(\sqrt{\frac{p_n}{n}}\right)O(n)=O_p(\sqrt{n}p_n).$$

Now, we analyse the stochastic terms with arguments from Wang (2011). We analysis the expectation for both terms in a small enough neighborhood, as stated above. The estimated rate implies the statement.

$$E_n^{[2]}(\beta_n)=\sum_{i=1}^{n}X_i^\intercal\Delta_i(\beta_n)A_i^{\frac{1}{2}}(\beta_n)R_i^{-1}G_i^{[2]}(\beta_n)\mathrm{diag}(y_i-\mu_i^0)X_i$$

$$= \sum_{i=1}^{n}\sum_{j=1}^{m_i}(y_{ij} - \mu^0_{ij})X_i^\intercal \Delta_i(\beta_n)A_i^{\frac{1}{2}}(\beta_n)R_i^{-1}G_i^{[2]}(\beta_n)e_j e_j^\intercal X_i$$

Write

$$\mathrm{E}[||E_n^{[2]}(\beta_n)||^2] = \sum_{i=1}^{n}\sum_{j_1=1}^{m_i}\sum_{j_2=1}^{m_i}\mathrm{E}[(y_{ij_1} - \mu^0_{ij_1})(y_{ij_2} - \mu^0_{ij_2})]$$

$$\mathrm{trace}(X_i^\intercal \Delta_i(\beta_n)A_i^{\frac{1}{2}}(\beta_n)R_i^{-1}G_i^{[2]}(\beta_n)e_{j_1}e_{j_1}^\intercal X_i X_i^\intercal e_{j_2}e_{j_2}^\intercal G_i^{[2]}(\beta_n)R_i^{-1}A_i^{\frac{1}{2}}(\beta_n)\Delta_i(\beta_n)X_i)$$

$$+ \sum_{i\neq l}\sum_{\text{correlated}}\sum_{j_1=1}^{m_i}\sum_{j_2=1}^{m_l}\mathrm{E}[(y_{ij_1} - \mu^0_{ij_1})(y_{lj_2} - \mu^0_{lj_2})]$$

$$\mathrm{trace}(X_i^\intercal \Delta_i(\beta_n)A_i^{\frac{1}{2}}(\beta_n)R_i^{-1}G_i^{[2]}(\beta_n)e_{j_1}e_{j_1}^\intercal X_i X_l^\intercal e_{j_2}e_{j_2}^\intercal G_l^{[2]}(\beta_n)R_l^{-1}A_l^{\frac{1}{2}}(\beta_n)\Delta_l(\beta_n)X_l)$$

$$=: I_1 + I_2.$$

We estimate

$$I_1 \leqslant C\sum_{i=1}^{n}\sum_{j_1=1}^{m_i}\sum_{j_2=1}^{m_i}|e_{j_1}^\intercal X_i X_i^\intercal e_{j_2}e_{j_2}^\intercal G_i^{[2]}(\beta_n)R_i^{-1}A_i^{\frac{1}{2}}(\beta_n)\Delta_i(\beta_n)X_i X_i^\intercal \Delta_i(\beta_n)A_i^{\frac{1}{2}}(\beta_n)R_i^{-1}G_i^{[2]}(\beta_n)e_{j_1}|.$$

We can bound

$$|e_{j_1}^\intercal X_i X_i^\intercal e_{j_2}e_{j_2}^\intercal G_i^{[2]}(\beta_n)R_i^{-1}A_i^{\frac{1}{2}}(\beta_n)\Delta_i(\beta_n)X_i X_i^\intercal \Delta_i(\beta_n)A_i^{\frac{1}{2}}(\beta_n)R_i^{-1}G_i^{[2]}(\beta_n)e_{j_1}|$$

$$\leqslant ||e_{j_1}^\intercal X_i||\; ||X_i^\intercal e_{j_2}||\; ||e_{j_2}^\intercal G_i^{[2]}(\beta_n)R_i^{-1}A_i^{\frac{1}{2}}(\beta_n)\Delta_i(\beta_n)X_i||\; ||X_i^\intercal \Delta_i(\beta_n)A_i^{\frac{1}{2}}(\beta_n)R_i^{-1}G_i^{[2]}(\beta_n)e_{j_1}||.$$

Now we have $||e_{j_1}^\intercal X_i|| \leqslant \sup_{i,j}||X_{ij}||$, $||X_i^\intercal e_{j_2}|| \leqslant \sup_{i,j}||X_{ij}||$,

$$||e_{j_2}^\intercal G_i^{[2]}(\beta_n)R_i^{-1}A_i^{\frac{1}{2}}(\beta_n)\Delta_i(\beta_n)X_i|| \leqslant C\ \mathrm{trace}(X_i X_i^\intercal)^{\frac{1}{2}}$$

and

$$||X_i^\intercal \Delta_i(\beta_n)A_i^{\frac{1}{2}}(\beta_n)R_i^{-1}G_i^{[2]}(\beta_n)e_{j_1}|| \leqslant C\ \mathrm{trace}(X_i X_i^\intercal)^{\frac{1}{2}}$$

leading to

$$I_1 \leqslant C\sup_{i,j}||X_{ij}||\sup_{i,j}||X_{ij}||\mathrm{trace}\left(\sum_{i=1}^{n}X_i X_i^\intercal\right) = O(p_n)O(np_n) = O(np_n^2).$$

Using the same arguments and Assumption A8, we can estimate

$$I_2 \leqslant C \sup_{i,j} ||X_{ij}|| \sup_{i,j} ||X_{ij}|| O(np_n) = O(p_n)O(p_n)O(n) = O(np_n^2).$$

With similar arguments, overall

$$\mathrm{E}[||E_n^{[1]}(\beta_n)||^2] = O(p_n)O(np_n) = O(np_n^2).$$

Thus, the proof is complete.

□

**Lemma 0.4.**

$$\mathrm{E}[||g_n(\beta_{n0}||^2] = O(np_n).$$

*Proof.* By definition,

$$\mathrm{E}[||g_n(\beta_{n0})||^2] = \sum_{i=1}^{n} \mathrm{E}\Big[\varepsilon_i^{\intercal}(\beta_{n0}) R_i^{-1} A_i^{\frac{1}{2}}(\beta_{n0}) \Delta_i(\beta_{n0}) X_i X_i^{\intercal} \Delta_i(\beta_{n0}) A_i^{\frac{1}{2}}(\beta_{n0}) R_i^{-1} \varepsilon_i(\beta_{n0})\Big]$$

$$+ \sum_{i \neq j \text{ correlated}} \mathrm{E}\Big[\varepsilon_j^{\intercal}(\beta_{n0}) R_j^{-1} A_j^{\frac{1}{2}}(\beta_{n0}) \Delta_j(\beta_{n0}) X_j X_i^{\intercal} \Delta_i(\beta_{n0}) A_i^{\frac{1}{2}}(\beta_{n0}) R_i^{-1} \varepsilon_i(\beta_{n0})\Big].$$

By Assumption A8, the number of clusters correlated with a specific cluster $i$ is bounded by some constant. This implies

$$\mathrm{E}[||g_n(\beta_{n0})||^2] \leqslant C \sum_{i=1}^{n} \sum_{j=1}^{m_i} X_{ij}^{\intercal} X_{ij} = O(np_n).$$

□

**Lemma 0.5.**

$$\lambda_{min}(M_n(\beta_{n0})) \geqslant C \lambda_{min}\Big(\sum_{i=1}^{n} X_i^{\intercal} X_i\Big)$$

*Proof.* Choose $b_n \in \mathbb{R}^{p_n}$ with $||b_n|| = 1$, then

$$b_n^\intercal M_n(\beta_{n0})b_n \geqslant \lambda_{\min}(\bar{R}) \min_i \lambda^2_{\min}(R_i^{-1}) \min_i \lambda_{\min}(A_i(\beta_{n0})) \min_i \lambda^2_{\min}(\Delta_i(\beta_{n0}))b_n^\intercal\left(\sum_{i=1}^n X_i^\intercal X_i\right)b_n$$

$$\geqslant C\lambda_{\min}\left(\sum_{i=1}^n X_i^\intercal X_i\right),$$

which implies the statement using Assumption A2.

□

*Proof of Proposition 3.* Let $b_n \in \mathbb{R}^{p_n}$ with $||b_n|| = 1$, $S_n := b_n^\intercal g_n(\beta_{n0}) = \sum_{i=1}^n b_n^\intercal g_{ni}(\beta_{n0})$ and $\sigma_n^2 := \mathrm{Var}(S_n) = b_n^\intercal M_n(\beta_{n0})b_n$. It is sufficient to show $S_n/\sqrt{\sigma_n^2} \to N(0,1)$ as $n \to \infty$ in distribution.

We follow the strategy as in Lumley and Mayer Hamblett (2003) and show $\lim_{n\to\infty} \mathrm{E}[(i\lambda - \bar{S}_n)e^{i\lambda\bar{S}_n}] = 0$, where $\bar{S}_n = \frac{S_n}{\sqrt{\mathrm{Var}(S_n)}}$. Since $\sup_n \mathrm{E}[\bar{S}_n^2] < \infty$, the results follows from Lemma 2 in [4].

Introduce $S_{i,n} := \sum_{i,j \text{ correlated}} b_n^\intercal g_{nj}(\beta_{n0})$ and $\bar{S}_{i,n}$ analogously. Write

$$(i\lambda - \bar{S}_n)e^{i\lambda\bar{S}_n} = i\lambda e^{i\lambda\bar{S}_n}(1 - \sigma_n^{-2}\sum_{i=1}^n b_n^\intercal g_{ni}S_{i,n}) - \sigma_n^{-1}e^{i\lambda\bar{S}_n}\sum_{i=1}^n b_n^\intercal g_{ni}(\beta_{n0})(1 - i\lambda\bar{S}_{i,n} - e^{-i\lambda\bar{S}_{i,n}})$$

$$-\sigma_n^{-1}\sum_{i=1}^n b_n^\intercal g_{ni}(\beta_{n0})e^{i\lambda(\bar{S}_n - \bar{S}_{i,n})} = A_1 - A_2 - A_3.$$

As in Lumley and Mayer Hamblett (2003), we calculate

$$\mathrm{E}[|A_1|^2] = \lambda^2\mathrm{E}[(1-\sigma_n^{-2}\sum_{i=1}^n b_n^\intercal g_{ni}(\beta_{n0})S_{i,n})^2] = \mathrm{Var}(\sigma_n^{-2}\sum_{i=1}^n b_n^\intercal g_{ni}(\beta_{n0})S_{i,n}) \leqslant \frac{1}{\sigma_n^4}4nM^3 \sup_i ||b_n^\intercal g_{ni}(\beta_{n0})||_4^4.$$

For fixed $i$, by Cauchy-Schwarz

$$[b_n^\intercal g_{ni}(\beta_{n0})]^2 \leqslant ||\varepsilon_i(\beta_{n0})||^2 \lambda_{\max}(X_i^\intercal X_i),$$

leading to

$$\mathrm{E}[[b_n^\intercal g_{ni}(\beta_{n0})]^4] = O(p_n^2)$$

and therefore

$$\mathrm{E}[|A_1|^2] \to 0 \text{ as } n \to \infty.$$

In addition,

$$\mathrm{E}[A_3] = 0,$$

since $b_n^\intercal g_{ni}(\beta_{n0})$ and $\bar{S}_n - \bar{S}_{i,n}$ are independent by definition.

As in Lumley and Mayer Hamblett (2003),

$$\mathrm{E}[|A_2|] \leqslant C\sigma_n^{-1}\mathrm{E}\left[\left|\sum_{i=1}^{n} b_n^\intercal g_{ni}(\beta_{n0})\right|\right]\mathrm{E}[\sup_i \bar{S}_{i,n}^2].$$

We find

$$\mathrm{E}[|A_2|] = C\sigma_n^{-3}O(\sqrt{n}p_n^{\frac{3}{2}}) \to 0 \text{ as } n \to \infty,$$

which concludes the statement. □

*Proof of Proposition 4.* Fix $\alpha_n \in \mathbb{R}^{p_n}$ with $||\alpha_n|| = 1$, write

$$\alpha_n^\intercal M_n^{-\frac{1}{2}}(\beta_{n0})g_n(\beta_{n0}) = \alpha_n^\intercal M_n^{-\frac{1}{2}}(\beta_{n0})g_n(\hat{\beta}_n) + \alpha_n^\intercal M_n^{-\frac{1}{2}}(\beta_{n0})G_n(\beta_n^*)(\hat{\beta}_n - \beta_{n0})$$

$$= \alpha_n^\intercal M_n^{-\frac{1}{2}}(\beta_{n0})H_n(\beta_{n0})(\hat{\beta}_n - \beta_{n0}) + \alpha_n^\intercal M_n^{-\frac{1}{2}}(\beta_{n0})\Big[G_n(\beta_n^*) - H_n(\beta_{n0})\Big](\hat{\beta}_n - \beta_{n0}),$$

where $\beta_n^*$ lies between $\hat{\beta}_n$ and $\beta_{n0}$.

Decompose

$$\alpha_n^\intercal M_n^{-\frac{1}{2}}(\beta_{n0})\Big[G_n(\beta_n) - H_n(\beta_{n0})\Big](\hat{\beta}_n - \beta_{n0})$$

$$= \alpha_n^\intercal M_n^{-\frac{1}{2}}(\beta_{n0})\Big[G_n(\beta_n) - H_n(\beta_n)\Big](\hat{\beta}_n - \beta_{n0}) + \alpha_n^\intercal M_n^{-\frac{1}{2}}(\beta_{n0})\Big[H_n(\beta_n) - H_n(\beta_{n0})\Big](\hat{\beta}_n - \beta_{n0}).$$

We have

$$\sup_{||\beta_n - \beta_{n0}|| \leqslant \Delta\sqrt{\frac{p_n}{n}}} |\alpha_n^\intercal M_n^{-\frac{1}{2}}(\beta_{n0})\Big[H_n(\beta_n) - H_n(\beta_{n0})\Big](\hat{\beta}_n - \beta_{n0})|$$

$$\leqslant \sup_{||\beta_n - \beta_{n0}|| \leqslant \Delta\sqrt{\frac{p_n}{n}}} ||\hat{\beta}_n - \beta_{n0}||\left(\alpha_n^\intercal M_n^{-\frac{1}{2}}(\beta_{n0})\Big[H_n(\beta_n) - H_n(\beta_{n0})\Big]\Big[H_n(\beta_n) - H_n(\beta_{n0})\Big]^\intercal M_n^{-\frac{1}{2}}(\beta_{n0})\alpha_n\right)^{\frac{1}{2}}$$

$$= O_p\left(\sqrt{\frac{p_n}{n}}\right) O\left(\frac{1}{\sqrt{n}}\right) O_p(p_n\sqrt{n}) = O_p\left(\frac{p_n^{\frac{3}{2}}}{\sqrt{n}}\right) = o_p(1),$$

by Remark 2 in Wang (2011), Lemma 0.2 and Lemma 0.5.

Applying the same arguments and Lemma 0.3 to the first term, we can conclude the statement.

□

*Proof of Proposition 5.* We use the same strategy as in Wang (2011) and write

$$\hat{\Sigma}_n - \Sigma_n = I_{n1} + I_{n2} + I_{n3},$$

with

$$I_{n1} = H_n^{-1}(\hat{\beta}_n)[\hat{M}_n(\hat{\beta}_n) - M_n(\beta_{n0})]H_n^{-1}(\hat{\beta}_n),$$

$$I_{n2} = [H_n^{-1}(\hat{\beta}_n) - H_n^{-1}(\beta_{n0})]M_n(\beta_{n0})H_n^{-1}(\hat{\beta}_n)$$

and

$$I_{n3} = H_n^{-1}(\hat{\beta}_{n0})M_n(\beta_{n0})[H_n^{-1}(\hat{\beta}_n) - H_n^{-1}(\beta_{n0})].$$

As in Wang (2011),

$$\sup_{||b_n||=1} |b_n^\intercal I_{n1} b_n|$$
$$\leqslant \frac{\max(|\lambda_{\max}(\hat{M}_n(\hat{\beta}_n) - M_n(\beta_{n0}))|, |\lambda_{\min}(\hat{M}_n(\hat{\beta}_n) - M_n(\beta_{n0}))|)}{\lambda_{\min}^2(H_n(\hat{\beta}_n))}.$$

Write, for $c_n \in \mathbb{R}^{p_n}$ with $||c_n|| = 1$,

$$|c_n^\intercal[\hat{M}_n(\hat{\beta}_n) - M_n(\beta_{n0})]c_n| \leqslant |c_n^\intercal[\hat{M}_n(\hat{\beta}_n) - \hat{M}_n(\beta_{n0})]c_n| + |c_n^\intercal[\hat{M}_n(\beta_{n0}) - M_n(\beta_{n0})]c_n|.$$

Consider the first term:

$$\sup_{||c_n||=1} |c_n^\intercal[\hat{M}_n(\hat{\beta}_n) - \hat{M}_n(\beta_{n0})]c_n| \leqslant \sup_{||c_n||=1} J_{n1} + \sup_{||c_n||=1} J_{n2} + \sup_{||c_n||=1} J_{n3} + \sup_{||c_n||=1} J_{n1}^c + \sup_{||c_n||=1} J_{n2}^c + \sup_{||c_n||=1} J_{n3}^c,$$

where

$$J_{n1} = \left| \sum_{i=1}^{n} c_n^\intercal X_i^\intercal [\Delta_i(\hat{\beta}_n) A_i^{\frac{1}{2}}(\hat{\beta}_n) - \Delta_i(\beta_{n0}) A_i^{\frac{1}{2}}(\beta_{n0})] R_i^{-1} \varepsilon_i(\hat{\beta}_n) \varepsilon_i^\intercal(\hat{\beta}_n) R_i^{-1} A_i^{\frac{1}{2}}(\hat{\beta}_n) \Delta_i(\hat{\beta}_n) X_i c_n \right|,$$

$$J_{n2} = \left| \sum_{i=1}^{n} c_n^\intercal X_i^\intercal \Delta_i(\beta_{n0}) A_i^{\frac{1}{2}}(\beta_{n0}) R_i^{-1} \varepsilon_i(\hat{\beta}_n) \varepsilon_i^\intercal(\hat{\beta}_n) R_i^{-1} [\Delta_i(\hat{\beta}_n) A_i^{\frac{1}{2}}(\hat{\beta}_n) - \Delta_i(\beta_{n0}) A_i^{\frac{1}{2}}(\beta_{n0})] X_i c_n \right|,$$

$$J_{n3} = \left| \sum_{i=1}^{n} c_n^\intercal X_i^\intercal \Delta_i(\beta_{n0}) A_i^{\frac{1}{2}}(\beta_{n0}) R_i^{-1} [\varepsilon_i(\hat{\beta}_n) \varepsilon_i^\intercal(\hat{\beta}_n) - \varepsilon_i(\beta_{n0}) \varepsilon_i^\intercal(\beta_{n0})] R_i^{-1} A_i^{\frac{1}{2}}(\beta_{n0}) \Delta_i(\beta_{n0}) X_i c_n \right|,$$

$$J_{n1}^c = \left| \sum_{i \neq j \text{ correlated}} c_n^\intercal X_i^\intercal [\Delta_i(\hat{\beta}_n) A_i^{\frac{1}{2}}(\hat{\beta}_n) - \Delta_i(\beta_{n0}) A_i^{\frac{1}{2}}(\beta_{n0})] R_i^{-1} \varepsilon_i(\hat{\beta}_n) \varepsilon_j^\intercal(\hat{\beta}_n) R_j^{-1} A_j^{\frac{1}{2}}(\hat{\beta}_n) \Delta_j(\hat{\beta}_n) X_j c_n \right|,$$

$$J_{n2}^c = \left| \sum_{i \neq j \text{ correlated}} c_n^\intercal X_i^\intercal \Delta_i(\beta_{n0}) A_i^{\frac{1}{2}}(\beta_{n0}) R_i^{-1} \varepsilon_i(\hat{\beta}_n) \varepsilon_j^\intercal(\hat{\beta}_n) R_j^{-1} [\Delta_j(\hat{\beta}_n) A_j^{\frac{1}{2}}(\hat{\beta}_n) - \Delta_j(\beta_{n0}) A_j^{\frac{1}{2}}(\beta_{n0})] X_j c_n \right|$$

and

$$J_{n3}^c = \left| \sum_{i \neq j \text{ correlated}} c_n^\intercal X_i^\intercal \Delta_i(\beta_{n0}) A_i^{\frac{1}{2}}(\beta_{n0}) R_i^{-1} [\varepsilon_i(\hat{\beta}_n) \varepsilon_j^\intercal(\hat{\beta}_n) - \varepsilon_i(\beta_{n0}) \varepsilon_j^\intercal(\beta_{n0})] R_j^{-1} A_j^{\frac{1}{2}}(\beta_{n0}) \Delta_j(\beta_{n0}) X_j c_n \right|.$$

We can estimate

$$J_{n1} \leqslant \sup_{||c_n||=1} \sum_{i=1}^{n} ||c_n^\intercal X_i^\intercal \left[ \Delta_i(\hat{\beta}_n) A_i^{\frac{1}{2}}(\hat{\beta}_n) - \Delta_i(\beta_{n0}) A_i^{\frac{1}{2}}(\beta_{n0}) \right] || \; ||R_i^{-1} \varepsilon_i(\hat{\beta}_n)||^2 \; ||A_i^{\frac{1}{2}}(\hat{\beta}_n) \Delta_i(\hat{\beta}_n) X_i c_n||$$

We have

$$||A_i^{\frac{1}{2}}(\hat{\beta}_n) \Delta_i(\hat{\beta}_n) X_i c_n|| \leqslant C ||X_i c_n||, \quad ||R_i^{-1} \varepsilon_i(\hat{\beta}_n)||^2 = O_p(1)$$

by Assumption A4 and

$$\left|\left| c_n^\intercal X_i^\intercal \left[ \Delta_i(\hat{\beta}_n) A_i^{\frac{1}{2}}(\hat{\beta}_n) - \Delta_i(\beta_{n0}) A_i^{\frac{1}{2}}(\beta_{n0}) \right] \right|\right| \leqslant C \sup_{ij} ||X_{ij}|| \; ||\hat{\beta}_n - \beta_{n0}|| \; ||X_i c_n||.$$

Thus,

$$J_{n1} = O(\sqrt{p_n})O_p\left(\frac{\sqrt{p_n}}{\sqrt{n}}\right)O(n) = O_p(p_n\sqrt{n}) = o_p(n).$$

The term $J_{n2}$ can be treated with the same arguments.

For $J_{n1}^c$ and $J_{n2}^c$ we can show analogously

$$J_{n1}^c = O_p(1)O(\sqrt{p_n})O_p\left(\frac{\sqrt{p_n}}{\sqrt{n}}\right)O(np_n) = O_p(p_n^2\sqrt{n}) = o_p(n)$$

and

$$J_{n2}^c = O_p(1)O(\sqrt{p_n})O_p\left(\frac{\sqrt{p_n}}{\sqrt{n}}\right)O(np_n) = O_p(p_n^2\sqrt{n}) = o_p(n)$$

We have

$$J_{n3} \leqslant \sup_{||c_n||=1} |\sum_{i=1}^n c_n^\intercal X_i^\intercal \Delta_i(\beta_{n0})A_i^{\frac{1}{2}}(\beta_{n0})R_i^{-1}[\varepsilon_i(\hat\beta_n)\varepsilon_i^\intercal(\hat\beta_n) - \varepsilon_i(\beta_{n0})\varepsilon_i^\intercal(\beta_{n0})]R_i^{-1}A_i^{\frac{1}{2}}(\beta_{n0})\Delta_i(\beta_{n0})X_i c_n|.$$

$$\leqslant \sup_{||c_n||=1}\sum_{i=1}^n ||c_n^\intercal X_i^\intercal \Delta_i(\beta_{n0})A_i^{\frac{1}{2}}(\beta_{n0})||\ ||R_i^{-1}||\ ||\varepsilon_i(\hat\beta_n)\varepsilon_i^\intercal(\hat\beta_n) - \varepsilon_i(\beta_{n0})\varepsilon_i^\intercal(\beta_{n0})||\ ||R_i^{-1}||\ ||A_i^{\frac{1}{2}}(\beta_{n0})\Delta_i(\beta_{n0})X_i c_n||$$

$$\leqslant C \sup_{||c_n||=1}\sum_{i=1}^n ||\varepsilon_i(\hat\beta_n)\varepsilon_i^\intercal(\hat\beta_n) - \varepsilon_i(\beta_{n0})\varepsilon_i^\intercal(\beta_{n0})||\ ||X_i c_n||^2.$$

We can write

$$||\varepsilon_i(\hat\beta_n)\varepsilon_i^\intercal(\hat\beta_n) - \varepsilon_i(\beta_{n0})\varepsilon_i^\intercal(\beta_{n0})||^2 = \sum_{k=1}^{m_i}\sum_{l=1}^{m_i}(\varepsilon_{ik}(\hat\beta_n)\varepsilon_{il}(\hat\beta_n) - \varepsilon_{ik}(\beta_{n0})\varepsilon_{il}(\beta_{n0}))^2.$$

Denote $A_{ik}(\beta_{n0})$ by $A_{ik}^0$.

$$\left|\frac{(y_{ik}-\mu_{ik}(\hat\beta_n))(y_{il}-\mu_{il}(\hat\beta_n))}{\sqrt{A_{ik}(\hat\beta_n)A_{il}(\hat\beta_n)}} - \frac{(y_{ik}-\mu_{ik}^0)(y_{il}-\mu_{il}^0)}{\sqrt{A_{ik}^0A_{il}^0}}\right|$$

$$\leqslant \left|\frac{(y_{ik}-\mu_{ik}(\hat\beta_n))(y_{il}-\mu_{il}(\hat\beta_n)) - (y_{ik}-\mu_{ik}^0)(y_{il}-\mu_{il}^0)}{\sqrt{A_{ik}^0A_{il}^0}}\right| + \left|\frac{(y_{ik}-\mu_{ik}(\hat\beta_n))(y_{il}-\mu_{il}(\hat\beta_n))}{\sqrt{A_{ik}^0A_{il}^0}}\delta_{kl}\right| =: I_{1kl}+I_{2kl},$$

where

$$\delta_{kl}(\beta_n) := \frac{\sqrt{A_{ik}^0A_{il}^0}}{\sqrt{A_{ik}(\hat\beta_n)A_{il}(\hat\beta_n)}} - 1.$$

Therefore,

$$||\varepsilon_i(\hat{\beta}_n)\varepsilon_i^{\intercal}(\hat{\beta}_n) - \varepsilon_i^0\varepsilon_i^{T0}||^2 \leqslant 2\sum_{k=1}^{m_i}\sum_{l=1}^{m_i} I_{1kl}^2 + 2\sum_{k=1}^{m_i}\sum_{l=1}^{m_i} I_{2kl}^2 =: I_1 + I_2.$$

Furthermore,

$$I_{1kl} \leqslant \frac{|(\mu_{ik}^0 - \mu_{ik}(\hat{\beta}_n))(\mu_{il}^0 - \mu_{il}(\hat{\beta}_n))|}{\sqrt{A_{ik}^0 A_{il}^0}} + \frac{|(y_{ik} - \mu_{ik}^0)(\mu_{il}^0 - \mu_{il}(\hat{\beta}_n))|}{\sqrt{A_{ik}^0 A_{il}^0}} + \frac{|(\mu_{ik}^0 - \mu_{ik}(\hat{\beta}_n))(y_{il} - \mu_{il}^0)|}{\sqrt{A_{ik}^0 A_{il}^0}} =: I_{11kl} + I_{12kl} + I_{13kl}$$

and thus

$$I_1 \leqslant 6\sum_{k=1}^{m_i}\sum_{l=1}^{m_i} I_{11kl}^2 + 6\sum_{k=1}^{m_i}\sum_{l=1}^{m_i} I_{12kl}^2 + 6\sum_{k=1}^{m_i}\sum_{l=1}^{m_i} I_{13kl}^2 =: I_{11} + I_{12} + I_{13}$$

We have

$$\frac{\partial \mu_{ik}}{\partial \beta_n}(\beta_n) = \mu_{ik}^{'}(\beta_n) X_{ik}$$

leading to, by Assumption A1,

$$\sum_{k=1}^{m_i} \frac{(\mu_{ik}^0 - \mu_{ik}(\hat{\beta}_n))^2}{A_{ik}^0} = \sum_{k=1}^{m_i} \frac{\mu_{ik}^{'2}(\tilde{\beta}_n)(\hat{\beta}_n - \beta_{n0})^{\intercal} X_{ik} X_{ik}^{\intercal}(\hat{\beta}_n - \beta_{n0})}{A_{ik}^0}$$

$$\leqslant CO(p_n)||\hat{\beta}_n - \beta_{n0}||^2 = O_p\left(\frac{p_n^2}{n}\right).$$

This leads to

$$I_{11} = O_p\left(\frac{p_n^4}{n^2}\right).$$

With the same argumentation, we get $I_{12} = O_p\left(\frac{p_n^2}{n}\right)$ and $I_{13} = O_p\left(\frac{p_n^2}{n}\right)$, by Assumption A4.

Now,

$$I_2 \leqslant 2O_p(1)\sum_{k=1}^{m_i}\sum_{l=1}^{m_i} \delta_{kl}^2(\beta_n).$$

Define $g(\beta_n) = \frac{(A_{ik}^0 A_{il}^0)^{\frac{1}{2}}}{(A_{ik}(\beta_n)A_{il}(\beta_n))^{\frac{1}{2}}}$. Then, we have $\delta_{kl}(\beta_n) = g(\hat{\beta}_n) - g(\beta_{n0})$ and

$$\frac{\partial g(\beta_n)}{\partial \beta_n} = \left(-\frac{1}{2}\right)\frac{(A_{ik}^0 A_{il}^0)^{\frac{1}{2}}}{(A_{ik}(\beta_n)A_{il}(\beta_n))^{\frac{3}{2}}}[A_{ik}^{'}(\beta_n)A_{il}(\beta_n)X_{ik} + A_{ik}(\beta_n)A_{il}^{'}(\beta_n)X_{il}].$$

With the same argumentation as in Wang (2011), we conclude

$$I_2 \leqslant C||\hat{\beta}_n - \beta_{n0}||^2 O(p_n) = O_p\left(\frac{p_n^2}{n}\right).$$

This concludes $J_{n3} = o_p(n)$ and, analoguously, $J_{n3}^c = o_p(n)$.

Next, we need to show:

$$\sup_{||c_n||=1} |c_n^\intercal [\hat{M}_n(\hat{\beta}_{n0}) - M_n(\beta_{n0})] c_n| = o_p(n).$$

First, note that

$$\mathrm{E}[c_n^\intercal \hat{M}_n(\hat{\beta}_{n0}) c_n] = c_n^\intercal M_n(\beta_{n0}) c_n.$$

In addition,

$$\mathrm{Var}\left(\frac{1}{n} c_n^\intercal \hat{M}_n(\beta_{n0}) c_n\right) \to 0 \text{ as } n \to \infty,$$

by the same estimates as in the proof of Proposition 3. This concludes the proof of the statement.

$\sup_{||b_n||=1} |b_n^\intercal I_{ni} b_n| = o_p(n^{-1})$ for $i = 2, 3$ follows completely by the same argumentation as in Wang (2011). □

## References


Lange, Christoph and Laird, Nan M. (2002). Power calculations for a general class of family-based association tests: dichotomous traits. *The American Journal of Human Genetics* **71**(3), 575–584.

Lumley, Thomas and Mayer Hamblett, Nicole. (2003). Asymptotics for marginal generalized linear models with sparse correlations.

Peyrot, Wouter J, Boomsma, Dorret I, Penninx, Brenda WJH and Wray, Naomi R. (2016). Disease and polygenic architecture: avoid trio design and appropriately account for unscreened control subjects for common disease. *The American Journal of Human Genetics* **98**(2), 382–391.

WANG, LAN. (2011). Gee analysis of clustered binary data with diverging number of covariates. *The Annals of Statistics* **39**(1), 389–417.

XIE, MINGE AND YANG, YANING. (2003). Asymptotics for generalized estimating equations with large cluster sizes. *The Annals of Statistics* **31**(1), 310–347.

YANG, JIAN, WEEDON, MICHAEL N, PURCELL, SHAUN, LETTRE, GUILLAUME, ESTRADA, KAROL, WILLER, CRISTEN J, SMITH, ALBERT V, INGELSSON, ERIK, O'CONNELL, JEFFREY R, MANGINO, MASSIMO *and others*. (2011). Genomic inflation factors under polygenic inheritance. *European Journal of Human Genetics* **19**(7), 807–812.

[]
